# Changes in electrocardiogram parameters during acute nonshivering cold exposure and associations with brown adipose tissue activity, plasma catecholamine levels, and brachial blood pressure in healthy adults

**DOI:** 10.14814/phy2.14718

**Published:** 2021-02-13

**Authors:** Juho R. H. Raiko, Teemu Saari, Janne Orava, Nina Savisto, Riitta Parkkola, Merja Haaparanta‐Solin, Pirjo Nuutila, Kirsi A. Virtanen

**Affiliations:** ^1^ Turku PET Centre Turku University Hospital Turku Finland; ^2^ Turku PET Centre University of Turku Turku Finland; ^3^ Department of Radiology Turku University Hospital and University of Turku Turku Finland; ^4^ Department of Endocrinology Turku University Hospital Turku Finland

**Keywords:** blood pressure, brown adipose tissue, catecholamine, cold exposure, electrocardiography, positron emission tomography

## Abstract

**Background:**

Sympathetic activity causes changes in electrocardiogram (ECG) during cold exposure and the changes have been studied mostly during hypothermia and less during mild acute nonshivering cold exposure. Cold‐induced sympathetic activity also activates brown adipose tissue (BAT) and increases arterial blood pressure (BP) and plasma catecholamine levels. We examined changes in ECG parameters during acute nonshivering cold exposure and their associations with markers of sympathetic activity during cold exposure: brachial blood pressure (BP), plasma catecholamine levels, and BAT activity measured by positron emission tomography (PET).

**Methods and results:**

Healthy subjects (M/F = 13/24, aged 20–55 years) were imaged with [^15^O]H_2_O (perfusion, N = 37) and [^18^F]FTHA to measure plasma nonesterified fatty acid uptake (NEFA uptake, *N* = 37) during 2‐h nonshivering cold exposure. 12‐lead ECG (*N* = 37), plasma catecholamine levels (*N* = 17), and brachial BP (*N* = 31) were measured at rest in room temperature (RT) and re‐measured after a 2‐h nonshivering cold exposure. There were significant differences between RT and cold exposure in P axis (35.6 ± 26.4 vs. 50.8 ± 22.7 degrees, *p* = 0.005), PR interval (177.7 ± 24.6 ms vs.163.0 ± 28.7 ms, *p* = 0.001), QRS axis (42.1 ± 31.3 vs. 56.9 ± 24.1, *p* = 0.003), and QT (411.7 ± 25.5 ms vs. 434.5 ± 39.3 ms, *p* = 0.001). There was no significant change in HR, QRS duration, QTc, JTc, and T axis during cold exposure. Systolic BP (127.2 ± 15.7 vs. 131.8 ± 17.9 mmHg, *p* = 0.008), diastolic BP (81.7 ± 12.0 vs. 85.4 ± 13.0 mmHg, *p* = 0.02), and plasma noradrenaline level increased during cold exposure (1.97 ± 0.61 vs. 5.07 ± 1.32 µmol/L, *p* = 0.001). Cold‐induced changes in ECG parameters did not correlate with changes in BAT activity, brachial BP, plasma catecholamines, or skin temperature.

**Conclusions:**

During short‐term nonshivering cold exposure, there were increases in P axis, PR interval, QRS axis, and QT compared to RT in healthy adults. Cold‐induced changes in ECG parameters did not correlate with BAT activity, brachial BP, or plasma catecholamine levels which were used as markers of cold‐induced sympathetic activity.

## Graphical abstract

Sympathetic activity causes changes in electrocardiogram (ECG) during cold exposure and the changes have been studied mostly during hypothermia and less during mild acute nonshivering cold exposure. Cold‐induced sympathetic activity also activates brown adipose tissue (BAT) and increases arterial blood pressure (BP) and plasma catecholamine levels. We examined changes in ECG parameters during acute nonshivering cold exposure and their associations with markers of sympathetic activity during cold exposure: brachial blood pressure (BP), plasma catecholamine levels and BAT activity measured by positron‐emission tomography (PET). During short‐term nonshivering cold exposure, there were increases in P axis, PR interval, QRS axis and QT compared to RT in healthy adults. Cold‐induced changes in ECG parameters did not correlate with BAT activity, brachial BP or plasma catecholamine levels which were used as markers of cold‐induced sympathetic activity.

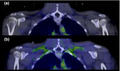

## 1 INTRODUCTION

Cold exposure is known to induce changes in the electrocardiogram (ECG). Short‐term cold exposure results in higher T‐wave amplitudes and shortening of QTc (Hintsala et al., 2014), while cold pressor test results in heart rate increase and shortening of QT interval correlating with simultaneous cold‐induced sympathetic activity (Doytchinova et al., 2017). Acute cold exposure also activates nonshivering thermogenesis in brown adipose tissue (BAT) due to sympathetic activation mediated by catecholamines (Zhu et al., 2014). Catecholamines adrenaline and noradrenaline participate in sympathetic cardiac regulation during cold exposure.

During hypothermia (core temperature <35°C), common ECG features are tremor artifact from shivering, initial increase in heart rate (HR) in mild hypothermia and decreased HR in more severe hypothermia, J waves, bradycardias, and prolongation of PR, QRS, and QT intervals (Drake & Flowers, 1980; Gould, 1985; Mareedu et al., 2008). Rapid immersion in cold water can trigger the autonomically mediated cold shock response which includes tachycardia, peripheral vasoconstriction, and hypertension (Tipton, 1989) and the coactivation of the sympathetic and parasympathetic autonomic nervous system can produce especially supraventricular and nodal cardiac arrhythmias (Datta & Tipton, 2006; Tipton et al., 1994). However, few studies have examined the effects of acute mild nonshivering cold exposure most studies focusing on hypothermia.

Sympathetic activity increases during cold exposure (Leppäluoto et al., 2005) resulting in higher HR and blood pressure (BP) (Hanna, 1999) which may contribute to the observed peak in cardiovascular morbidity and mortality during colder months (Pell & Cobbe, 1999) although the link between sympathetic nervous system activity and mortality during cold periods remains speculative. Higher incidence of both arrhythmias (Anand et al., 2007; Frost et al., 2002) and acute myocardial infarctions have been observed during colder periods (Jia et al., 2012).

As BAT activity and arterial blood pressure are regulated by the sympathetic nervous system, we find it interesting if cold‐induced BAT activity, plasma catecholamine levels, and brachial blood pressure would correlate with ECG parameters measured in cold due to vascular resistance and the chronotropic, inotropic, dromotropic, and lusitropic effects of catecholamines on the heart. Thus, subjects with high BAT activity might share a similar ECG phenotype. Cold‐induced increase in BAT activity and BP could be considered as surrogate markers of sympathetic activity during cold exposure, while plasma catecholamine levels would act as a more direct marker of sympathetic activity. BAT is activated by elevated plasma catecholamine levels in humans (Wang et al., 2011) and catecholamines also increase the number of brown adipocytes and upregulates uncoupling protein 1 expression in BAT (Cannon & Nedergaard, 2004), which would support the hypothesis of potential correlations between BAT activity and ECG parameters associated with sympathetic activity.

Our study hypothesis is that ECG parameters associated with sympathetic activity (i.e., HR, conduction and repolarization times) would alter during acute nonshivering cold exposure in healthy adults and associate with surrogate markers of cold‐induced sympathetic activity such as supraclavicular BAT activity and brachial BP and also with cold‐induced changes in plasma catecholamine levels. In the current study, we examined the effects of acute 2‐h mild nonshivering cold exposure on conventional resting 12‐lead ECG parameters in healthy adults compared to ECG measurements performed in room temperature. Additionally, we studied associations between cold‐induced changes in ECG parameters and supraclavicular BAT activity measured with positron emission tomography (PET) in room temperature (RT) and during nonshivering cold exposure. The effect of mild superficial cold exposure on cardiac electrical function in healthy adults is not well established as most previous studies have examined ECG changes in severe whole‐body cold exposure and hypothermia. Thus, our study also provides insight into changes in cardiac electrophysiology in healthy adults during acute nonshivering cold exposure which can, from a clinical standpoint, alter ECG parameters in clinical patients exposed to mild superficial cooling prior to acquiring ECGs. Additionally, since BAT‐activating interventions are researched as potential treatments for obesity and diabetes, associations between BAT activity and ECG parameters may possess further clinical significance.

## 2 MATERIALS AND METHODS

### 2.1 Ethical approval

Prior to acceptance in the study, the subjects gave written informed consent and the studies conformed to the standards set by the latest revision of the Declaration of Helsinki. The study was approved by the properly constituted local medical ethics committee of the Southwestern Finland Medical District (the ethical review approval number: T231/2013) and the project has been registered in the Clinical Trials Database (NCT01985503).

The cohort consisted of study subjects who participated in research projects into BAT activity in healthy adults (U Din, 2018). The measured ECG parameters and their associations with BAT activity, plasma catecholamines, and brachial BP have not been previously reported elsewhere.

**FIGURE 1:**
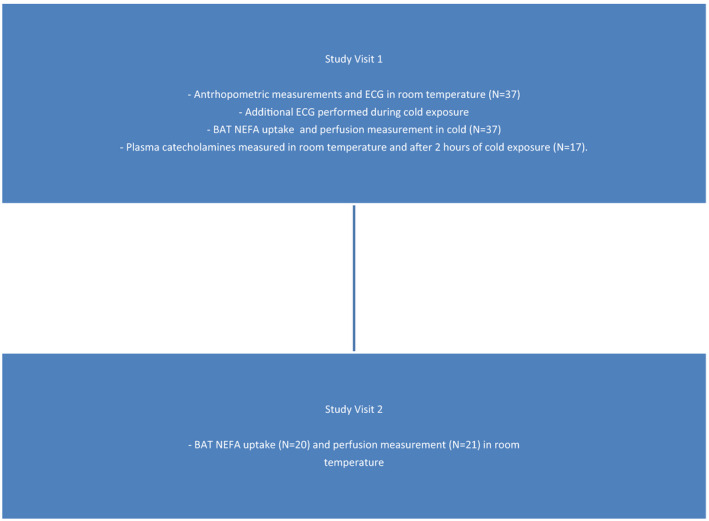
Description of the study structure. All subjects underwent ECG measurement in room temperature, while ECG in cold stimulus was performed only in subjects imaged with [^18^F]FTHA.

The study subjects were recruited with newspaper adverts. Subjects were required to be nondiabetic, non‐hypertensive and without a history of cardiovascular disease. Exclusion criteria were diabetes, hypertension, dyslipidemia, history of cardiovascular disease and thyroid dysfunction, and pregnancy or breastfeeding prior to acceptance in the study. Subjects with abnormal ECG changes (ischemia, bundle branch blocks, AV blocks) were excluded from the study. The study cohort consisted of 22–55‐year‐old healthy subjects (*N* = 37, M/F 13/24). Study structure is shown in Figure 1.

ECG measurements used in our analyses were performed during Study Visit 1 as shown in Figure 1. The study subjects came to the research center at 8 AM and had been instructed to avoid strenuous physical exercise on the day prior and during the morning of Study Visits 1 and 2. The study subjects lay in supine position on a bed for a period of around 10 min before the acquisition of the resting the 12‐lead conventional ECG measurement at RT. This was performed prior to the setting of intravenous connection to avoid the potential effect from the pain of the procedure on ECG parameters. Skin was wiped clean with alcohol and body hair was shaved on the site of the leads when necessary. Additionally, 12‐lead ECG measurements were performed after 2 h of cold exposure in supine position and the cooling continued during the ECG recording. During Study Visit 2, only the PET scan in RT was performed.

Plasma catecholamine levels were drawn from a venous catheter in room temperature prior to the cold exposure and after the 2‐h cold exposure in 17 study subjects as described previously (U Din, 2018). Additionally, blood pressure was measured with an Omron blood pressure monitor (Omron Healthcare Inc., US) in 31 subjects in RT and then after 2 h of cold exposure. Brachial BP was measured during Study Visit 1 first in RT after a 10 min period in the supine position, and then, after the 2 h cold exposure. Single measurements from the right arm (blood sampling venous cannula was in the left arm) were used to minimize the effect of consecutive BP measurements on BP and sympathetic activity. BP measurements were performed after the acquisition of ECG. Cooling started once all measurements in RT were performed and cooling was stopped once all measurements at 120 min of cooling were performed.

The study subjects lay in the supine position throughout the period between the ECG measured in RT and the ECG measured after the cooling. The ECG recordings were 10 s in duration. The recordings were checked for technical quality and segments without baseline variation from breathing, muscle movement or tremor or other technical artifacts were selected for analysis. P axis, PR interval, QRS axis, QRS duration, QT, QTc, JTc, and T axis were measured manually by a single researcher. The values were averaged from three heartbeats. The end of the T‐wave was determined with a tangent drawn to the steepest last limb of the T‐wave and the end of the T‐wave was the intersection of this tangent and the baseline.

Healthy adult subjects underwent positron emission tomography (PET) with [^18^F]FTHA and [^15^O]H_2_O (*N* = 37) to measure nonesterified fatty acid (NEFA) uptake and perfusion in the supraclavicular BAT depot during cold exposure (U Din, 2018). Intravenous injection of [^18^F]FTHA was given and a dynamic emission scan was performed (frames: 1 × 60 s, 6 × 30 s, 1 × 60 s, 3 × 300 s, 2 × 600 s). Nonshivering cold exposure with adjustable cooling blankets had started 2 h prior to and continued during the PET imaging and 20 subjects were reimaged at RT with [^15^O]H_2_O and [^18^F]FTHA to measure nonstimulated BAT metabolism. All PET scans were performed after an overnight fast. The study subject lay on a bed with one cooling blanket below them and the other placed on top of them. The cooling blanket under the study subject reached form the feet to the thoracic region and the blanket placed on the subject reached from the feet to the abdomen. The temperature of the cooling blanket was adjusted to prevent muscle tremor and the temperature of the cooling blanket was recorded at 5 min intervals during the cooling to calculate the mean cooling temperature. The cooling blanket included plastic tubing where cooled water was circulated and the water temperature was increased if muscle tremor started to appear as described previously (U Din, 2018). The study subjects wore underpants and light pyjama‐like patient clothing with long sleeves and pants during the cold exposure. Skin temperature during cooling was measured with a contact thermometer attached laterally on the abdominal region midway between the iliac crest and the lowest ribs on the sight side of the study subject. Skin temperature was recorded at 5 min intervals and the skin temperature at the start of the cooling was used as skin temperature in RT and skin temperature at 120 min of cooling was used as the skin temperature after cold exposure. The study subjects were in supine position during the measurements in RT and during cold exposure.

A constant room temperature was maintained during the RT studies (mean ± SD 22.5 ± 0.4°C).

QTc was calculated as follows: QTc = QT/√RR interval.

JTc was calculated as follows: JTc = QTc − QRS duration.

PET images were analyzed with Carimas 2.8. imaging analysis software (Turku PET Centre, University of Turku).

### 2.2 Statistical analysis

The results are expressed as the mean group values with standard deviations (SD). Spearman's correlation was used to measure bivariate correlations between variables. Paired two‐way *t*‐test was used to examine differences in mean levels between RT and cold exposure. We calculated the changes in parameters from baseline in RT to measurements performed during cold exposure and then analyzed correlations between changes in ECG parameters and BAT activity, plasma catecholamine levels, and brachial BP to assess if there were simultaneous cold‐induced changes in these variables. Due to the large number of variables in our correlation analyses, Bonferroni correction was used to assess a critical P‐value corrected for multiple comparisons. SPSS 23 (IBM) was used as the statistical software.

## 3 RESULTS

### 3.1 Description of cohort characteristics

The study cohort characteristics are displayed in Table 1. Mean age was 38.4 ± 9.9 years and mean BMI was 27.4 ± 4.6 kg/m^2^. Mean ECG parameters in RT were within normal range. Mean cold‐activated BAT NEFA uptake in cold in the cohort (*N* = 37) was 0.77 ± 0.49 μmol/100 g/min, and BAT perfusion was 19.1 ± 17.5 ml/100 g/min (Table 1 displays the mean values of subjects who were PET imaged both at RT and during cold exposure, *N* = 20).

**TABLE 1:** Description of study cohort

Variable	Room temperature Mean±SD	Cold exposure Mean±SD	P for comparison
Age	38.4 ± 9.9	NA	NA
Males/Females (*N*)	13/24	NA	NA
HR at rest (1/min)	60.5 ± 6.6	59.7 ± 7.3	0.42
P axis (degrees)	35.6 ± 26.4	50.8 ± 22.7	0.005
PR interval (ms)	177.7 ± 24.6	163.0 ± 28.7	0.001
QRS duration (ms)	91.±10.2	89.4 ± 11.8	0.31
QRS axis (degrees)	42.1 ± 31.3	56.9 ± 24.1	0.003
QT (ms)	411.7 ± 25.5	434.5 ± 39.3	0.001
QTc (ms)	423.9 ± 26.4	426.8 ± 31.0	0.52
JTc (ms)	333.8 ± 28.8	337.4 ± 36.3	0.35
T axis (degrees)	27.6 ± 18.9	43.3 ± 20.7	0.07
Waist (cm)	93.4 ± 16.0	NA	NA
BMI (kg/m^2^)	27.4 ± 4.6	NA	NA
Systolic BP (mmHg)	127.2 ± 15.7	131.8 ± 17.9	0.008
Diastolic BP (mmHg)	81.7 ± 12.0	85.4 ± 13.0	0.02
Plasma adrenaline (µmol/L)	0.169 ± 0.044	0.176 ± 0.081	0.74
Plasma noradrenaline (µmol/L)	1.97 ± 0.61	5.07 ± 1.32	0.001
BAT NEFA uptake (umol/100 g/min)	0.67 ± 0.70	1.15 ± 1.65^a^	0.19
BAT perfusion (ml/100 g/min)	11.0 ± 11.1	16.0 ± 14.5^a^	0.08
Mean cooling temperature (°C)	NA	7.2 ± 2.6	NA
Skin temperature (°C)	32.2 ± 1.6	32.0 ± 2.0	0.48

^a^ Includes only subjects with PET scans performed both at RT and during cold exposure.

### 3.2 Cold‐induced changes in ECG parameters, BAT activity, brachial BP, and plasma catecholamines

Table 1 displays the comparison of ECG parameters between baseline measurements in RT and measurements performed during cold exposure to examine the potential effects of cold‐induced changes. Differences between RT and cold exposure were significant in P axis (35.6 ± 26.4 vs. 50.8 ± 22.7°, *p* = 0.005), (PR interval (177.7 ± 24.6 ms vs.163.0 ± 28.7 ms, *p* = 0.001), QRS axis (42.1 ± 31.3 vs. 56.9 ± 24.1, *p* = 0.003), and QT (411.7 ± 25.5 ms vs. 434.5 ± 39.3 ms, *p* = 0.001). There was no significant difference in HR, QRS duration, QTc, JTc, and T axis.

**FIGURE 2:**
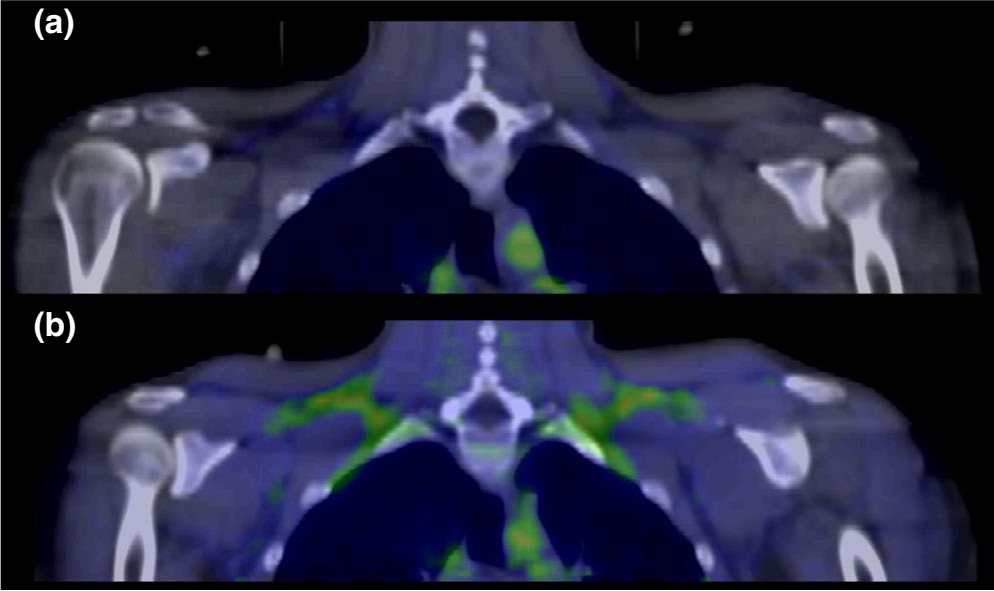
Baseline supraclavicular BAT perfusion in room temperature during fasting (a) and elevated BAT perfusion during cold exposure in the same subject (b). Highest tracer activity is marked with green in the PET/CT fusion images.

Systolic (127.2 ± 15.7 vs. 131.8 ± 17.9 mmHg, *p* = 0.008) and diastolic BP (81.7 ± 12.0 vs. 85.4 ± 13.0 mmHg, *p* = 0.02) both increased during cold exposure. BAT NEFA uptake did not increase significantly in cold (0.67 ± 0.70 vs. 1.15 ± 1.65 µmol/100 g/min, *p* = 0.19) and increase in BAT perfusion was also nonsignificant (11.0 ± 11.1 vs. 16.0 ± 14.5 ml/100 g/min, *p* = 0.08). Figure 2 shows the cold‐induced increase in supraclavicular BAT compared to RT in a PET/CT image.

Cold exposure induced a significant increase in plasma noradrenaline level (1.97 ± 0.61 vs. 5.07 ± 1.32 µmol/L, *p* = 0.0001), while plasma adrenaline level did not change (*p* = 0.74). Although brachial BP and noradrenaline level increased suggesting a significant sympathetic response to our cooling method, skin surface temperature did not change significantly during the cold exposure (32.2 ± 1.6 vs. 32.0 ± 2.0°C, *p* = 0.48).

### 3.3 Correlations between cold‐induced changes in ECG parameters and BAT activity, brachial BP, and plasma catecholamines

Table 2 shows the correlations between cold‐induced changes in ECG parameters and BAT activity, BP, and plasma catecholamines. We observed no significant correlations between the examined variables.

**TABLE 2:** Correlation between cold‐induced changes in ECG parameters and BAT activity, plasma catecholamines and brachial BP. Only ECG parameters with significant changes during cold exposure were included

Variables	ΔPR interval	ΔP axis	ΔQRS axis	ΔQT
ΔBAT NEFA uptake	−0.233 (*p* = 0.37)	−0.132 (*p* = 0.76)	−0.014 (*p* = 0.96)	−0.394 (*p* = 0.12)
ΔBAT perfusion	−0.100 (*p* = 0.68)	−0.347 (*p* = 0.40)	−0.203 (*p* = 0.40)	−0.068 (*p* = 0.78)
ΔNoradrenaline	−0.189 (*p* = 0.48)	−0.230 (*p* = 0.42)	−0.435 (*p* = 0.09)	−0.097 (*p* = 0.72)
ΔAdrenaline	−0.189 (*p* = 0.48)	0.301 (*p* = 0.30)	−0.435 (*p* = 0.09)	0.013 (*p* = 0.96)
ΔSystolic blood pressure	0.155 (*p* = 0.57)	0.140 (*p* = 0.61)	−0.066 (*p* = 0.81)	−0.241 (*p* = 0.37)
ΔDiastolic blood pressure	0.190 (*p* = 0.50)	0.059 (*p* = 0.78)	−0.052 (*p* = 0.79)	−0.242 (*p* = 0.20)

All variables are the absolute changes from RT measurements to measurements performed during cold exposure. Bonferroni's adjusted critical *p* < 0.002.

### 3.4 Correlations between ECG parameters and BAT activity, brachial BP, and plasma catecholamines in room temperature

Correlations between ECG parameters and BAT activity, BP, plasma catecholamines, and skin temperature were examined in Table 3. BAT NEFA uptake in RT correlated significantly with QRS axis (r = 0.456, *p* = 0.04) and JTc (r = 0.500, *p* = 0.03). Additionally, plasma noradrenaline level correlated with HR (r = 0.687, *p* = 0.002). However, all correlations exceeded the Bonferroni's corrected critical *p* < 0.001.

**TABLE 3:** Correlation between ECG parameters and BAT activity, plasma catecholamines and brachial BP in room temperature (RT)

Variables	HR	PR interval	P axis	QRS duration	QRS axis	QT	QTc	JTc	T axis
BAT NEFA uptake in RT	0.202 (*p* = 0.39)	−0.016 (*p* = 0.95)	−0.151 (*p* = 0.62)	−0.368 (*p* = 0.11)	0.456 (*p* = 0.04)	0.158 (*p* = 0.51)	0.409 (*p* = 0.07)	0.500 (*p* = 0.025)	0.309 (*p* = 0.31)
BAT perfusion in RT	0.048 (*p* = 0.84)	−0.089 (*p* = 0.70)	−0.209 (*p* = 0.49)	−0.313 (*p* = 0.17)	0.271 (*p* = 0.23)	0.111 (*p* = 0.63)	0.125 (*p* = 0.59)	0.281 (*p* = 0.22)	0.231 (*p* = 0.30)
Noradrenaline in RT	0.687 (*p* = 0.002)	−0.467 (*p* = 0.06)	0.371 (*p* = 0.47)	0.056 (*p* = 0.83)	−0.023 (*p* = 0.93)	−0.204 (*p* = 0.43)	0.440 (*p* = 0.08)	0.304 (*p* = 0.24)	0.087 (*p* = 0.87)
Adrenaline in RT	0.251 (*p* = 0.33)	−0.123 (*p* = 0.64)	−0.143 (*p* = 0.79)	0.139 (*p* = 0.60)	−0.046 (*p* = 0.86)	−0.072 (*p* = 0.78)	0.183 (*p* = 0.48)	0.018 (*p* = 0.95)	−0.377 (*p* = 0.46)
Systolic blood pressure in RT	0.044 (*p* = 0.80)	0.232 (*p* = 0.18)	−0.471 (*p* = 0.09)	0.067 (*p* = 0.70)	0.068 (*p* = 0.69)	0.133 (*p* = 0.45)	0.123 (*p* = 0.48)	0.086 (*p* = 0.62)	0.126 (*p* = 0.67)
Diastolic blood pressure in RT	−0.054 (*p* = 0.76)	0.219 (*p* = 0.21)	0.070 (*p* = 0.81)	−0.078 (*p* = 0.66)	0.095 (*p* = 0.59)	−0.117 (*p* = 0.50)	−0.234 (*p* = 0.18)	−0.202 (*p* = 0.24)	0.199 (*p* = 0.49)
Skin temperature in RT	−0.097 (*p* = 0.59)	0.139 (*p* = 0.43)	−0.506 (*p* = 0.07)	0.033 (*p* = 0.85)	0.009 (*p* = 0.96)	−0.079 (*p* = 0.65)	−0.210 (*p* = 0.23)	−0.162 (*p* = 0.35)	−0.161 (*p* = 0.36)

Bonferroni's adjusted critical *p* < 0.001.

### 3.5 Correlations between ECG parameters and BAT activity, brachial BP, and plasma catecholamines during cold exposure

In Table 4, we examined correlations between ECG measurements and BAT activity, brachial BP, plasma catecholamines, skin temperature, and mean cooling temperature during cold exposure. HR correlated with systolic BP (r = 0.382, *p* = 0.03) and skin temperature (r = −0.423, *p* = 0.04). These correlations nonetheless exceeded the Bonferroni's corrected critical *p* < 0.001.

**TABLE 4:** Correlation between ECG parameters and BAT activity, plasma catecholamines, brachial BP, skin temperature, and mean cooling temperature during cold exposure

Variables	HR	PR interval	P axis	QRS duration	QRS axis	QT	QTc	JTc	T axis
BAT NEFA uptake during cold exposure	0.210 (*p* = 0.23)	−0.205 (*p* = 0.25)	−0.095 (*p* = 0.63)	−0.205 (*p* = 0.25)	−0.259 (*p* = 0.14)	−0.073 (*p* = 0.68)	0.216 (*p* = 0.22)	0.272 (*p* = 0.12)	0.341 (*p* = 0.08)
BAT perfusion during cold exposure	0.176 (*p* = 0.33)	−0.233 (*p* = 0.19)	−0.240 (*p* = 0.19)	−0.105 (*p* = 0.56)	−0.198 (*p* = 0.27)	−0.121 (*p* = 0.50)	0.041 (*p* = 0.83)	0.238 (*p* = 0.18)	−0.075 (*p* = 0.71)
Noradrenaline during cold exposure	0.111 (*p* = 0.67)	−0.459 (*p* = 0.07)	−0.240 (*p* = 0.41)	−0.124 (*p* = 0.64)	0.034 (*p* = 0.90)	−0.209 (*p* = 0.42)	0.025 (*p* = 0.93)	0.115 (*p* = 0.67)	−0.024 (*p* = 0.94)
Adrenaline during cold exposure	0.183 (*p* = 0.48)	−0.375 (*p* = 0.14)	−0.183 (*p* = 0.53)	−0.055 (*p* = 0.83)	0.079 (*p* = 0.76)	0.158 (*p* = 0.55)	0.322 (*p* = 0.21)	0.313 (*p* = 0.22)	−0.257 (*p* = 0.37)
Systolic blood pressure during cold exposure	0.382 (*p* = 0.03)	0.127 (*p* = 0.50)	−0.025 (*p* = 0.90)	−0.213 (*p* = 0.25)	−0.007 (*p* = 0.97)	−0.294 (*p* = 0.11)	0.135 (*p* = 0.47)	0.138 (*p* = 0.46)	0.106 (*p* = 0.60)
Diastolic blood pressure during cold exposure	0.117 (*p* = 0.54)	0.181 (*p* = 0.34)	0.059 (*p* = 0.78)	−0.267 (*p* = 0.16)	−0.052 (*p* = 0.79)	−0.242 (*p* = 0.20)	−0.077 (*p* = 0.69)	0.007 (*p* = 0.97)	0.130 (*p* = 0.53)
Skin temperature after 2 h cold exposure	−0.423 (*p* = 0.04)	−0.102 (*p* = 0.59)	−0.011 (*p* = 0.96)	0.179 (*p* = 0.34)	0.332 (*p* = 0.07)	0.286 (*p* = 0.13)	−0.024 (*p* = 0.90)	−0.082 (*p* = 0.67)	0.044 (*p* = 0.84)
Mean cooling temperature between 60–120 min	0.034 (*p* = 0.84)	0.107 (*p* = 0.53)	0.203 (*p* = 0.17)	−0.080 (*p* = 0.64)	0.111 (*p* = 0.51)	0.052 (*p* = 0.76)	0.089 (*p* = 0.60)	0.077 (*p* = 0.65)	0.013 (*p* = 0.94)

Bonferroni's adjusted critical *p* < 0.001.

## 4 DISCUSSION

According to our hypothesis, acute nonshivering cold exposure would increase HR and prolong conduction and repolarization times in conventional resting ECG. In our results, P axis increased, PR interval decreased and QRS axis and QT increased significantly during cold exposure compared to RT. Our cold exposure method did not alter surface skin temperature but brachial BP and plasma noradrenaline level increased during cold exposure suggesting nonshivering cooling was sufficient to induce a measurable sympathetic response. Cold‐induced changes in BAT activation, brachial BP, and plasma catecholamine levels was expected to associate with changes in ECG parameters displaying chronotropy, cardiac conductance, and repolarization such as HR, QTc, and JTc. However, changes in these surrogate markers of cold‐stimulated sympathetic activity did not correlate with cold‐induced‐changes in ECG parameter levels. Neither was there any correlation between BAT activity level and ECG parameters in cold. This might suggest that the autonomic regulation of cardiac electrophysiology may be somewhat independent of cold‐stimulated BAT activity, brachial BP, and plasma catecholamine levels during mild acute cold exposure. However, nonstimulated BAT fatty acid metabolism in RT correlated directly with QRS axis and JTc. Additionally, plasma noradrenaline level correlated with HR in RT. HR in cold correlated with systolic BP and skin temperature during cold exposure. Additionally, the lack or presence of significant correlations in our study setting does not prove or disprove causal relationships between the examined variables since we only examined correlations.

Previous studies with severe cooling have shown prolonged conduction and repolarization times during hypothermia (Drake & Flowers, 1980; Gould, 1985; Mareedu et al., 2008). Our 2‐h mild nonshivering cold exposure (mean cooling temperature 7.2 ± 2.6°C) did not alter skin surface temperature and likely did not induce core hypothermia in our study subjects, and thus, our results would be comparable to previous studies using acute mild cold exposure not resulting in hypothermia. Hintsala et al., 2014 studied hypertensive men using a 15 min whole‐body exposure to −10°C air while wearing winter clothes and Doytchinova et al., (2017) used a cold water pressor test. There are no previous studies to our knowledge with a similar mild cold exposure using heat‐conducting cooling blankets in healthy adults as was performed in the current study. Acute cold exposure has resulted in increased HR and shortened QTc linked to sympathetic activity (Hintsala et al., 2014; Doytchinova et al., 2017). While we did not observe significant increase in HR, PR decreased, P and QRS axes tilted rightwards and QT elongated. Furthermore, HR‐dependent ECG parameters such as QT, JT, and PR may be affected by small variations in HR nondetectable in our study which can explain the observed effect of cold exposure on the ECG parameters not corrected for HR. Since brachial BP increased during cold exposure, the electrical axis of the heart would have been expected to tilt leftwards contrary to our findings. One potential mechanism affecting the observed rightward tilt in P and QRS axes during cold exposure might be a small increase in pulmonary arterial pressure during acute cold exposure which has been previously observed in rats (Kashimura, 1993), while a 20 min 16°C cold exposure in a cooling suit increased pulmonary capillary wedge pressure in humans (Wilson et al., 2007).

Our cooling method resulted in a significant increase in plasma noradrenaline, while plasma adrenaline level did not change. Adrenal medulla secretes catecholamines adrenaline and noradrenaline into circulation during certain stimuli such as cold exposure. Additionally, noradrenaline acts as a transmitter in the sympathetic nervous system. Plasma noradrenaline originates mostly from sympathetic nerves and the amount reaching the circulation before being metabolized varies depending on the nerve ending‐effector junctions in the tissue (Kopin et al., 1978). Previous studies on plasma catecholamine levels have shown that noradrenaline levels elevate more easily (Hiramatsu et al., 1984; O’Malley et al., 1984; Weeke & Gundersen, 1983) than plasma adrenaline levels which may increase or remain constant during cold exposure (Leppäluoto et al., 1988; Wagner et al., 1987).

Short cold exposure using a cooling blanket without immersion in cold water may not be sufficient to increase plasma adrenaline levels significantly as water immersion has been shown to elevate plasma adrenaline levels (Galbo et al., 1979). Additionally, our 2‐h cold exposure with cooling blankets may have been insufficient to induce a sufficient sympathetic response to increase catecholamine levels to such a level that would correlate with ECG parameters in cold. However, plasma noradrenaline correlated significantly with HR at RT suggesting that plasma catecholamine level did display sympathetic activity in our study at least in RT.

Mean skin temperature measured laterally from the abdominal region did not change significantly during cold exposure in our study. This may be due to our nonshivering cold exposure with adjusted temperature which aimed to avoid muscle activation in favor of BAT activation due to nonshivering thermogenesis. Potentially, a nonadjusted cold exposure with a constant temperature may have resulted in more cooling, and thus, higher sympathetic activity but the resulting skeletal muscle activation would have interfered with the measurement of supraclavicular BAT activity since the region is surrounded by skeletal muscles. However, skin temperature during cold exposure correlated negatively with HR in cold meaning that lower skin temperature associated with higher HR. Thus, skin temperature would act as a marker of achieved effective cooling since mean cooling temperature did not correlate with any ECG parameter.

Cold‐induced changes in brachial BP levels did not correlate with changes in ECG parameters during cold exposure in our study although we observed a significant increase in brachial BP during cooling. Short‐term whole‐body cold exposure has been previously shown to increase systolic and diastolic blood pressure via sympathetic activation (Gao et al., 2012; Greaney & Stanhewicz, 1985; Wilson et al., 1985). However, the blood pressure increase due to our short‐term nonshivering cold exposure may be insufficient to induce significant changes in ECG. Additionally, subjects with hypertension were excluded prior to acceptance in the study. The effect of cold‐induced increase in BAT perfusion on systemic vascular be can regarded insignificant as the amount of cold‐activated BAT is relatively low in adult humans (Virtanen et al., 2009).

Cold‐induced BAT activity measured by plasma fatty acid uptake and tissue perfusion did not correlate with changes in ECG measurements from RT to cold. However, nonstimulated BAT NEFA uptake in RT correlated positively with QRS axis and JTc which may be due to baseline level of sympathetic activity. JTc is a marker of ventricular repolarization corrected for QRS duration and the observed correlation would suggest that BAT activity might reflect cardiac repolarization at rest. Right axis deviation of QRS is usually associated with increased pulmonary BP but our cohort was screened for previous known disease and did not contain subjects with a diagnosed pulmonary disease. Additionally, high sympathetic activity at rest would increase systemic BP which would tilt QRS complex leftwards. However, BAT activity is higher in women and in young people and lower in men and the elderly and the latter group is more likely to have higher systemic BP which might explain the observed association in RT. The correlations between BAT activity and ECG parameters were attenuated when measurements from cold exposure were used. These findings would suggest that BAT activity does not function as a surrogate marker of sympathetic regulation of cardiac electrophysiology during cold exposure. Moreover, whole‐body cold exposure has been shown to induce a simultaneous sympathetic and parasympathetic activation (Mäkinen et al., 2008) which may act as a confounding factor also in our examined correlations as parasympathetic activity was not measured. This may contribute hypothetically to the lack of significant correlations between the cold‐induced changes in ECG and our surrogate markers of sympathetic activity.

Morbidity and mortality from causes such as cardiovascular disease increase during periods of cold weather (Basu & Samet, 2002). The suggested mechanisms behind the increased number in cardiovascular deaths include higher sympathetic activity predisposing to arrhythmias (Vaseghi & Shivkumar, 2008) and increased blood pressure due to increased HR and peripheral vascular resistance during cold exposure (Hanna, 1999). A cold test in healthy adults does not alter coronary blood flow but patients with an ischemic heart show a decrease in coronary blood flow in cold (Houdas et al., 1992). Therefore, ischemia‐related ECG changes would not be observed in our healthy adult cohort during cooling. Arrhytmias are also more common during colder periods as both the number of atrial fibrillations and ventricular arrhytmias peak during winter months (Anand et al., 2007; Frost et al., 2002). Prolonged QT can cause ventricular tachycardia and fibrillation (Vaseghi & Shivkumar, 2008). Subjects with an overactive sympathetic nervous system such as patients with hypertension are more prone to arrhythmias (Manolis et al., 2012) and further studies would be required to assess if high BAT activity in otherwise healthy individuals is linked with arrhythmias. JTc and QRS axis in RT were the only ECG parameters to associate with nonstimulated BAT activity and no correlations were found during cold exposure.

### 4.1 Limitations

ECG measurements are highly dependent on the placement of leads on the patient. In the current study, leads were attached by a medical professional. Inter‐patient variance in placement of leads on the chest wall should not affect our results since P axis, QRS axis, and T axis are determined by limb leads. Skin was wiped with alcohol prior to lead attachment to minimize resistance on the skin surface.

Nonshivering cold exposure is required in PET studies of BAT activity to avoid muscle tremor and the concomitant muscle activation interfering with estimation of BAT activity. Thus, we only performed a relatively short and mild cold exposure which also limits the observed effects on ECG parameters, plasma catecholamine levels, and brachial BP. This is reflected by the fact that skin temperature did not change significantly during our nonshivering cold exposure. During cold exposure, peripheral blood flow decreases and reduces heat transfer between the body core and more superficial tissues such as skin thus increasing insulation and preventing core heat loss. In cold water immersion, insulation begins to increase when skin temperature is below 35°C and becomes maximal when skin temperature is 31°C or below (Veicsteinas et al., 1982). The mean skin temperature in our study was 32.2°C at baseline in RT and 32.0°C after 2 h of nonshivering cold exposure using a cooling blanket and this may suggest that insulation due to vasoconstriction may have limited the effect of cold exposure in our study cohort.

Skin temperature did not decrease in our study during cold exposure which may be due to several factors. The chosen method of skin temperature measurement at a single site may have underestimated the cold stimulus and skin temperature should have been measured from several sites. Additionally, muscle tremor was estimated subjectively by the study subject or visually by the researcher. Thus, the cold stimulus may have been counter‐balanced by enhanced heat production in skeletal muscles, which would suggest that there may have been some shivering that could not be quantified for instance with EMG. Third, our cold stimulus may have been simply insufficient suggesting that the study findings may not have been caused by only the cold stimulus alone but may have been influenced by other confounding experimental variables not accounted for. Therefore, further research would be needed with skin temperature measurement from several sites, monitoring of muscle tremor with for instance EMG and perhaps a case‐control study with a sham condition to examine if the observed findings are caused the cold stimulus and not by confounding factors.

The mean cooling temperature of 7.2°C was calculated as the mean temperature of the cooling water circulated in the cooling blankets during the final hour of the 2 h cold exposure. We used two cooling blankets, one below and one on top of the study subject while the study subject wore a light pyjama‐type clothing. A previous study using a cooling suit with 10°C cold water perfusion achieved a decrease from 34.0 ± 0.02 to 27.2 ± 0.02°C in the mean skin temperature during a 2 h shivering cold exposure measuring mean skin temperature from the forehead, chest, biceps, forearm, abdomen, lower and upper back, front and back calf, quadriceps, hamstrings, and finger (Haman et al., (2002). In another study with a 2 h shivering cold exposure using a cooling suit, 18°C cold water perfusion resulted in a significant decrease in mean skin temperature measured from the forehead, chest, biceps, forearm, abdomen, lower and upper back, front and back calf, quadriceps, hamstrings, and hand (Blondin et al., 2017). Our study aimed to perform a nonshivering cold exposure to minimize muscle tremor which would interfere with ECG measurements and PET imaging of BAT. However, our cooling method using cooling blankets while the study subject wore a pyjama‐type clothing may have resulted in less heat transfer between the cooling blanket and the skin, and thus, our cooling method was less effective than a cooling suit without insulation from light clothing. Thus, future studies with cooling suits may be required.

The correlations between the absolute values of ECG parameters and the markers of sympathetic activity in cold do not imply that these variables would alter similarly during cold stress. Additionally, the observed correlation do not indicate that factors with significant correlations would have a causal relationship and the lack of correlation does not prove that ECG parameters and markers of sympathetic activity would be independent of one another. Therefore, the potential causal relationships we present are purely speculative and further studies are required to asses if any causality exists.

Due to the large number of correlations examined in the study, we used Bonferroni correction to correct critical *p*‐value for multiple comparisons. Thus, no *p*‐value were below the corrected critical *p*‐value. The large number of variables increases the likelihood of finding a significant correlation generated by random chance and we recognize this as a limitation in interpreting the findings in our study.

Plasma catecholamine levels were available only from 17 subjects which somewhat limits the power of the analyses. However, blood pressure levels were measured in 31 subjects out of 37 which can act as a surrogate of sympathetic activity during cold exposure.

Study subjects were screened for ST depression or elevation, T inversion and bundle branch or atrioventricular blocks prior to acceptance in the study and subjects with abnormal ECGs were excluded from the study. Additionally, none of our subjects had been diagnosed with a heart disease. Thus, associations between BAT activity and these ECG abnormalities cannot be examined in our study.

Due to the radiation dose acquired in PET CT imaging, our study cohort was relatively small for ethical reasons limiting the statistical power of our data set. However, we employed quantitative dynamic PET scanning instead of the commonly used semiquantitative static SUV PET scans for more accurate evaluation of BAT activity.

## 5 CONCLUSIONS

Nonshivering 2‐h cold exposure in healthy resulted in increased P axis, PR interval, QRS axis and QT compared to RT. Additionally, cold‐induced changes in BAT activity, brachial BP and plasma catecholamine levels did not correlate with cold‐induced changes in ECG parameters. However, brachial BP and plasma noradrenaline levels increased during our cold exposure suggesting our cold exposure was sufficient to induce sympathetic activation although mean skin temperature did not change during the 2‐h cooling at a mean temperature of 7.2 ± 2.6°C. However, the lack of correlations between our markers of sympathetic activity in cold and cold‐induced changes in ECG parameters would suggest that cardiac electrophysiology measured by ECG may be independent of cold‐induced changes in BAT activity, brachial BP, and plasma catecholamine levels during nonshivering cold exposure but further studies on potential causal relationships are required.

## CONFLICT OF INTEREST

We declare no conflict of interest.

## AUTHOR CONTRIBUTIONS

PN and KV participated in conception or design of the work. All authors participated in acquisition, analysis, or interpretation of data for the work and in drafting of the work or revising it critically for important intellectual content. All authors approved the final version of the manuscript and agree to be accountable for all aspects of the work in ensuring that questions related to the accuracy or integrity of any part of the work are appropriately investigated and resolved. All persons designated as authors qualify for authorship, and all those who qualify for authorship are listed.

## References

[phy214718-bib-0001] Anand, K. , Aryana, A. , Cloutier, D. , Hee, T. , Esterbrooks, D. , Mooss, A. N. , & Mohiuddin, S. M. (2007). Circadian, daily, and seasonal distributions of ventricular tachyarrhythmias in patients with implantable cardioverter‐defibrillators. American Journal of Cardiology, 100(7), 1134–1138. 10.1016/j.amjcard.2007.04.063 17884377

[phy214718-bib-0002] Basu, R. , & Samet, J. M. (2002). Relation between elevated ambient temperature and mortality: a review of the epidemiologic evidence. Epidemiologic Reviews, 24(2), 190–202. 12762092 10.1093/epirev/mxf007

[phy214718-bib-0003] Blondin, D. P. , Daoud, A. , Taylor, T. , Tingelstad, H. C. , Bézaire, V. , Richard, D. , Carpentier, A. C. , Taylor, A. W. , Harper, M. E. , Aguer, C. , & Haman, F. (2017). Four‐week cold acclimation in adult humans shifts uncoupling thermogenesis from skeletal muscles to brown adipose tissue. Journal of Physiology, 595(6), 2099–2113. 10.1113/JP273395 PMC5350439 28025824

[phy214718-bib-0004] Cannon, B. , & Nedergaard, J. (2004). Brown adipose tissue: function and physiological significance. Physiological Reviews, 84, 277–359. 14715917 10.1152/physrev.00015.2003

[phy214718-bib-0005] Datta, A. , & Tipton, M. (2006). Respiratory responses to cold water immersion: neural pathways, interactions, and clinical consequences awake and asleep. Journal of Applied Physiology, 100, 2057–2064. 16714416 10.1152/japplphysiol.01201.2005

[phy214718-bib-0006] Drake, C. E. , & Flowers, N. C. (1980). ECG changes in hypothermia from sepsis and unrelated to exposure. Chest, 77, 685–686. 7363691 10.1378/chest.77.5.685

[phy214718-bib-0007] Frost, L. , Paaske Johnsen, S. , Pedersen, L. , Husted, S. , Engholm, G. , Toft Sørensen, H. , & Rothman, K. J. (2002). Seasonal variation in hospital discharge diagnosis of atrial fibrillation: a population‐based study. Epidemiology, 13(2), 211–215. 11880763 10.1097/00001648-200203000-00017

[phy214718-bib-0008] Galbo, H. , Houston, M. E. , Christensen, N. J. , Holst, J. J. , Nielsen, B. , Nygaard, E. , & Suzuki, J. (1979). The effect of water temperature on the hormonal response to prolonged swimming. Acta Physiologica Scandinavica, 105(3), 326–337. 443063 10.1111/j.1748-1716.1979.tb06348.x

[phy214718-bib-0009] Gao, Z. , Wilson, T. E. , Drew, R. C. , Ettinger, J. , & Monahan, K. D. (2012). Altered coronary vascular control during cold stress in healthy older adults. American Journal of Physiology. Heart and Circulatory Physiology, 302(1), 312. 10.1152/ajpheart.00297.2011 PMC3334247 22003058

[phy214718-bib-0010] Gould, L. , Gopalaswamy, G. , Kim, B. S. , & Patel, C. (1985). The Osborn wave in hypothermia. Angiology, 36, 125–129. 4025922 10.1177/000331978503600210

[phy214718-bib-0011] Greaney, J. L. , Stanhewicz, A. E. , Kenney, W. L. , & Alexander, L. M. (1985). Muscle sympathetic nerve activity during cold stress and isometric exercise in healthy older adults. Journal of Applied Physiology, 117(6), 648–657. 10.1152/japplphysiol.00516.2014 PMC4157163 25103970

[phy214718-bib-0012] Haman, F. , Péronnet, F. , Kenny, G. P. , Massicotte, D. , Lavoie, C. , Scott, C. , & Weber, J. M. (2002). Effect of cold exposure on fuel utilization in humans: plasma glucose, muscle glycogen, and lipids. Journal of Applied Physiology (1985), 93(1), 77–84. 10.1152/japplphysiol.00773.2001 12070189

[phy214718-bib-0013] Hanna, J. M. (1999). Climate, altitude, and blood pressure. Human Biology, 71(4), 553–582. 10453102

[phy214718-bib-0014] Hintsala, H. , Kenttä, T. V. , Tulppo, M. , Kiviniemi, A. , Huikuri, H. V. , Mäntysaari, M. , Keinänen‐Kiukaannemi, S. , Bloigu, R. , Herzig, K.‐H. , Antikainen, R. , Rintamäki, H. , Jaakkola, J. J. K. , & Ikäheimo, T. M. (2014). Cardiac repolarization and autonomic regulation during short‐term cold exposure in hypertensive men: an experimental study. PLoS One, 9(7), e99973. 24983379 10.1371/journal.pone.0099973 PMC4077657

[phy214718-bib-0015] Hiramatsu, K. , Yamada, T. , & Katakura, M. (1984). Acute effects of cold on blood pressure, reninangiotensin‐aldosterone system, catecholamines and adrenal steroids in man. Clinical and Experimental Pharmacology and Physiology, 11(2), 171–179. 6378465 10.1111/j.1440-1681.1984.tb00254.x

[phy214718-bib-0016] Houdas, Y. , Deklunder, G. , & Lecroart, J. L. (1992). Cold exposure and ischemic heart disease. International Journal of Sports Medicine, 13(Suppl 1), S179–S181. 1483767 10.1055/s-2007-1024632

[phy214718-bib-0017] Jia, E.‐Z. , Xu, Z.‐X. , Cai, H.‐Z. , Guo, C. Y. , Li, L. I. , Zhu, T.‐B. , Wang, L.‐S. , Cao, K.‐J. , Ma, W.‐Z. , & Yang, Z.‐J. (2012). Time distribution of the onset of chest pain in subjects with acute ST‐elevation myocardial infarction: an eight‐year, single‐center study in China. PLoS One, 7(3), e32478. 22427844 10.1371/journal.pone.0032478 PMC3299668

[phy214718-bib-0018] Kashimura, O. (1993). Effects of acute exposure to cold on pulmonary arterial blood pressure in awake rats. Nihon Eiseigaku Zasshi., 48(4), 859–863. 8254993 10.1265/jjh.48.859

[phy214718-bib-0019] Kopin, I. J. , Lake, R. C. , & Ziegler, M. (1978). Plasma levels of norepinephrine. Annals of Internal Medicine, 88(5), 671–680. 646261 10.7326/0003-4819-88-5-671

[phy214718-bib-0020] Leppäluoto, J. , Korhonen, I. , Huttunen, P. , & Hassi, J. (1988). Serum levels of thyroid and adrenal hormones, testosterone, TSH, LH, GH and prolactin in men after a 2‐h stay in a cold room. Acta Physiologica Scandinavica, 132, 543–548. 3227893 10.1111/j.1748-1716.1988.tb08363.x

[phy214718-bib-0021] Leppäluoto, J. , Pääkkönen, T. , Korhonen, I. , & Hassi, J. (2005). Pituitary and autonomic responses to cold exposures in man. Acta Physiologica Scandinavica, 184(4), 255–264. 16026418 10.1111/j.1365-201X.2005.01464.x

[phy214718-bib-0022] Mäkinen, T. M. , Mäntysaari, M. , Pääkkönen, T. , Jokelainen, J. , Palinkas, L. A. , Hassi, J. , Leppäluoto, J. , Tahvanainen, K. , & Rintamäki, H. (2008). Autonomic nervous function during whole‐body cold exposure before and after cold acclimation. Aviation, Space and Environmental Medicine, 79(9), 875–882. 10.3357/asem.2235.2008 18785356

[phy214718-bib-0023] Manolis, A. J. , Rosei, E. A. , Coca, A. , Cifkova, R. , Erdine, S. E. , Kjeldsen, S. , Lip, G. Y. H. , Narkiewicz, K. , Parati, G. , Redon, J. , Schmieder, R. , Tsioufis, C. , & Mancia, G. (2012). Hypertension and atrial fibrillation: diagnostic approach, prevention and treatment. Position paper of the Working Group ‘Hypertension Arrhythmias and Thrombosis’ of the European Society of Hypertension. Journal of Hypertension, 30(2), 239–252. 22186358 10.1097/HJH.0b013e32834f03bf

[phy214718-bib-0024] Mareedu, R. K. , Grandhe, N. P. , Gangineni, S. , & Quinn, D. L. (2008). Classic EKG changes of hypothermia. Clin Med Res., 6(3–4), 107–108. 19325173 10.3121/cmr.2008.809 PMC2670529

[phy214718-bib-0025] O’Malley, B. P. , Cook, N. , Richardson, A. , Barnett, D. B. , & Rosenthal, F. D. (1984). Circulating catecholamine, thyrotrophin, thyroid hormone and prolactin responses of normal subjects to acute cold exposure. Clinical Endocrinology ‐ Oxford, 21(3), 285–291. 6478632 10.1111/j.1365-2265.1984.tb03471.x

[phy214718-bib-0026] Pell, J. P. , & Cobbe, S. M. (1999). Seasonal variations in coronary heart disease. QJM, 92(12), 689–696. 10581331 10.1093/qjmed/92.12.689

[phy214718-bib-0028] Tipton, M. J. (1989). The initial responses to cold‐water immersion in man. Clinical Science, 77, 581–588. 2691172 10.1042/cs0770581

[phy214718-bib-0029] Tipton, M. J. , Kelleher, P. C. , & Golden, F. S. (1994). Supraventricular arrhythmias following breath‐hold submersions in cold water. Undersea and Hyperbaric Medicine, 21, 305–313. 7950804

[phy214718-bib-0030] U Din, M. , Saari, T. , Raiko, J. , Kudomi, N. , Maurer, S. F. , Lahesmaa, M. , Fromme, T. , Amri, E.‐Z. , Klingenspor, M. , Solin, O. , Nuutila, P. , & Virtanen, K. A. (2018). Postprandial oxidative metabolism of human brown fat indicates thermogenesis. Cell Metabolism, 28(2), 207–216.e3. 29909972 10.1016/j.cmet.2018.05.020

[phy214718-bib-0031] Vaseghi, M. , & Shivkumar, K. (2008). The role of the autonomic nervous system in sudden cardiac death. Progress in Cardiovascular Diseases, 50(6), 404–419. 18474284 10.1016/j.pcad.2008.01.003 PMC2752648

[phy214718-bib-0032] Veicsteinas, A. , Ferretti, G. , & Rennie, D. W. (1982). Superficial shell insulation in resting and exercising men in cold water. Journal of Applied Physiology: Respiratory, Environmental and Exercise Physiology, 52(6), 1557–1564. 10.1152/jappl.1982.52.6.1557 7107465

[phy214718-bib-0033] Virtanen, K. A. , Lidell, M. E. , Orava, J. , Heglind, M. , Westergren, R. , Niemi, T. , Taittonen, M. , Laine, J. , Savisto, N.‐J. , Enerbäck, S. , & Nuutila, P. (2009). Functional brown adipose tissue in healthy adults. New England Journal of Medicine, 360(15), 1518–1525. 10.1056/NEJMoa0808949 19357407

[phy214718-bib-0034] Wagner, D. , Shen, C. , Salanova, V. , Meshberger, C. , Chen, L. S. , Kincaid, J. C. , Coffey, A. C. , Wu, G. , Li, Y. , Kovacs, R. J. , Everett, T. H. , Victor, R. , Cha, Y.‐M. , Lin, S.‐F. , & Chen, P.‐S. (2017). Simultaneous noninvasive recording of skin sympathetic nerve activity and electrocardiogram. Heart Rhythm: the Official Journal of the Heart Rhythm Society, 14(1), 25–33. 10.1016/j.hrthm.2016.09.019 PMC5182108 27670627

[phy214718-bib-0035] Wagner, J. A. , Horvath, S. M. , Kitagawa, K. , & Bolduan, N. W. (1987). Comparisons of blood and urinary responses to cold exposures in young and older men and women. Journals of Gerontology. Series A, Biological Sciences and Medical Sciences, 42(2), 173–179. 10.1093/geronj/42.2.173 3819343

[phy214718-bib-0036] Wang, Q. , Zhang, M. , Ning, G. , Gu, W. , Su, T. , Xu, M. , Li, B. , & Wang, W. (2011). Brown adipose tissue in humans is activated by elevated plasma catecholamines levels and is inversely related to central obesity. PLoS One, 6(6), e21006. 21701596 10.1371/journal.pone.0021006 PMC3118816

[phy214718-bib-0037] Weeke, J. , & Gundersen, H. J. (1983). The effect of heating and central cooling on serum TSH, GH, and norepinephrine in resting normal man. Acta Physiologica Scandinavica, 117(1), 33–39. 6858704 10.1111/j.1748-1716.1983.tb07176.x

[phy214718-bib-0038] Wilson, T. E. , Sauder, C. L. , Kearney, M. L. , Kuipers, N. T. , Leuenberger, U. A. , Monahan, K. D. , & Ray, C. A. (1985). Monahan KD & Ray CA (2007) Skin‐surface cooling elicits peripheral and visceral vasoconstriction in humans. Journal of Applied Physiology, 103(4), 1257–1262. 10.1152/japplphysiol.00401.2007 17673561

[phy214718-bib-0039] Wilson, T. E. , Tollund, C. , Yoshiga, C. C. , Dawson, E. A. , Nissen, P. , Secher, N. H. , & Crandall, C. G. (2007). Effects of heat and cold stress on central vascular pressure relationships during orthostasis in humans. Journal of Physiology, 585(Pt 1), 279–285. 10.1113/jphysiol.2007.137901 PMC2375461 17901119

[phy214718-bib-0040] Zhu, Z. , Spicer, E. G. , Gavini, C. K. , Goudjo‐Ako, A. J. , Novak, C. M. , & Shi, H. (2014). Enhanced sympathetic activity in mice with brown adipose tissue transplantation (transBATation). Physiology & Behavior, 125, 21–29. 24291381 10.1016/j.physbeh.2013.11.008 PMC3896387

